# Long non-coding RNA profiling links subgroup classification of endometrioid endometrial carcinomas with trithorax and polycomb complex aberrations

**DOI:** 10.18632/oncotarget.5399

**Published:** 2015-09-26

**Authors:** Yunyun Jiang, Gabriel G. Malouf, Jianping Zhang, Xiaofeng Zheng, Yunxin Chen, Erika J. Thompson, John N. Weinstein, Ying Yuan, Jean-Philippe Spano, Russell Broaddus, Nizar M. Tannir, David Khayat, Karen H. Lu, Xiaoping Su

**Affiliations:** ^1^ Department of Gynecologic Oncology and Reproductive Medicine, UT MD Anderson Cancer Center, Houston, TX, USA; ^2^ Department of Medical Oncology, Groupe Hospitalier Pitié-Salpêtrière, University Pierre and Marie Curie (Paris VI), Institut Universitaire de Cancérologie, AP-HP, Paris, France; ^3^ Department of Bioinformatics and Computational Biology, UT MD Anderson Cancer Center, Houston, TX, USA; ^4^ Department of Genitourinary Medical Oncology, UT MD Anderson Cancer Center, Houston, TX, USA; ^5^ Department of Genetics, UT MD Anderson Cancer Center, Houston, TX, USA; ^6^ Department of Biostatistics, UT MD Anderson Cancer Center, Houston, TX, USA; ^7^ Department of Pathology, UT MD Anderson Cancer Center, Houston, TX, USA

**Keywords:** endometrioid endometrial carcinoma, long non-coding RNA, RNA-Seq, expression profiling, polycomb complex

## Abstract

**Background:**

Integrative analysis of endometrioid endometrial carcinoma (EEC) using multiple platforms has distinguished four molecular subgroups. However, the landscape of expressed long non-coding RNAs (lncRNA) and their role in charting EEC subgroups and determining clinical aggressiveness remain largely unknown.

**Materials and Methods:**

We performed integrative analysis of lncRNAs in EEC using The Cancer Genome Atlas (TCGA) molecular RNAseq profiles of 191 primary tumors for which genomic data were also available. We established lncRNA subgroup classification, correlated it with chromatin modifying gene expression, and described correlations between our lncRNA classification and clinico-genomic tumor features.

**Results:**

Using stringent criteria, we identified 1,931 expressed lncRNAs and predicted potential drivers through integrative analysis. Unsupervised clustering of lncRNA expression revealed three robust categories: basal-like, luminal-like and *CTNNB1*-enriched subgroups. Basal-like subgroup was enriched for aggressive tumors with higher pathological grade (*p* < 0.0001), TNM stage (*p* = 0.01), and somatic mutations in trithorax-group genes (*MLL, MLL2* and *MLL3*); and it overexpressed polycomb genes *EZH2* and *CBX2*. In contrast to the luminal-like subgroup, progesterone (*PGR*) and estrogen receptor (*ESR1*) genes were highly down-regulated in the EEC basal-like subgroup. Consistent with its enrichment for *CTNNB1* mutations (69%), lncRNA profile of the *CTNNB1*-enriched EEC subgroup was highly similar to that of the *CTNNB1*-enriched liver cancer subgroup.

**Conclusions:**

Our results reveal the utility of systematic characterization of clinically annotated EEC in three clinically relevant subgroups. They also highlight the convergence of aberrations in polycomb- and trithorax-group genes in aggressive basal EEC subtypes, providing a rationale for further investigation of epigenetic therapy in this setting.

## BACKGROUND

Endometrial cancer (EC) arises from the tissue lining the uterus and is the most common gynecological malignancy in the United States [1]. The American Cancer Society estimated that 52,630 new EC cases and 8,590 related deaths occurred in 2014 [1]. EC is divided into two categories based upon histology: type I is endometrial endometrioid carcinoma (EEC), which accounts for ~80% of all uterine cancers [2]; type II includes serous and clear cell tumors, which are relatively rare.

EEC is generally associated with a better prognosis compared to that associated with type II EC. However, prognosis varies among patients with EEC, with FIGO stage being an established prognostic indicator [3]. Higher histologic grade is also associated with increased likelihood of disease recurrence or more advanced stage at diagnosis. Although many endometrial cancers are cured with surgery in combination with systematic chemotherapy and radiotherapy, the prognosis for patients with recurrent or advanced disease remains poor. Potential biomarkers have been identified and are being translated into clinical trials for drugs that target EEC [4]. However, these biomarkers have been identified in a retrospective manner and further validations in larger cohorts are warranted [4].

Significant efforts have been made to investigate the etiology of EC at the molecular level. Early studies using traditional immunohistochemistry and Sanger sequencing methods have identified mutations of *PTEN, KRAS* and *CTNNB1* genes [5]. The development of next-generation sequencing methods has allowed better characterization of EC using integrative analysis on different platforms [6]. For instance, the Cancer Genome Atlas (TCGA) study revealed hotspot mutations in the *POLE* gene in 7.3% of EC cases, which also harbored an ultramutated phenotype [6]. In addition to this ultramutated subclass, three additional molecular categories have been established by TCGA, as follows: microsatellite instability hypermutated, copy-number low and copy-number high subclasses [6]. Importantly, the majority of serous-like cancers belong to the copy-number high subclass, which displays a low mutation rate in addition to extensive somatic copy number alterations [6]. Although TCGA delved into the genomics of EC, allowing researchers to further decipher the landscape of alterations within hundreds of tumor cases, the portrait of expressed long non-coding RNA (lncRNA) in this disease remains unknown.

Initially considered as transcriptional noise, many thousands of non-coding RNAs are currently reported to be transcribed by the genome [7]. Integrative and mechanistic studies have begun to unravel the functions of those RNA, which are non-coding RNAs longer than 200bp [8]. Some studies have shown that lncRNAs act as epigenetic regulators through modifying the conformation of chromatins [8]. This process necessitates an interaction with polycomb and/or trithorax group proteins that regulate the nuclear organization of their target genes. This is the case for lncRNA *HOTAIR*, which reprograms chromatin states through coupling histone H3K27 methylation and H3K4 demethylation [9]. Importantly, a recent study has linked *HOTAIR* to poor prognosis in endometrial cancers [10]. However, to the best of our knowledge, systematic studies of lncRNA in a large cohort of women with EEC have not been reported.

For the first time, to our knowledge, we herein report the lncRNA subgroup classification of EEC through unsupervised clustering and integrative analysis of a large dataset of primary tumors. We intentionally focused exclusively in EEC and excluded serous-like EC, as EEC share similar pathological features and there is an urgent need to investigate the input of molecular classification of those tumors. We identified three lncRNAs subgroups that correlated with clinico-genomic tumor aberrations. Those include basal-like and luminal-like subgroups, which are reminiscent of breast cancer, as well as a third subgroup, namely *CTNNB1*-enriched, which is reminiscent of *CTNNB1*-enriched liver cancer. The application of lncRNA profiling can potentially be utilized as a tool for predicting clinical outcome and selecting targeted therapy.

## RESULTS

### Expressed long non-coding RNAs in endometrioid endometrial carcinomas are located in the vicinity of genes involved in histone H4 acetylation

**Figure 1:**
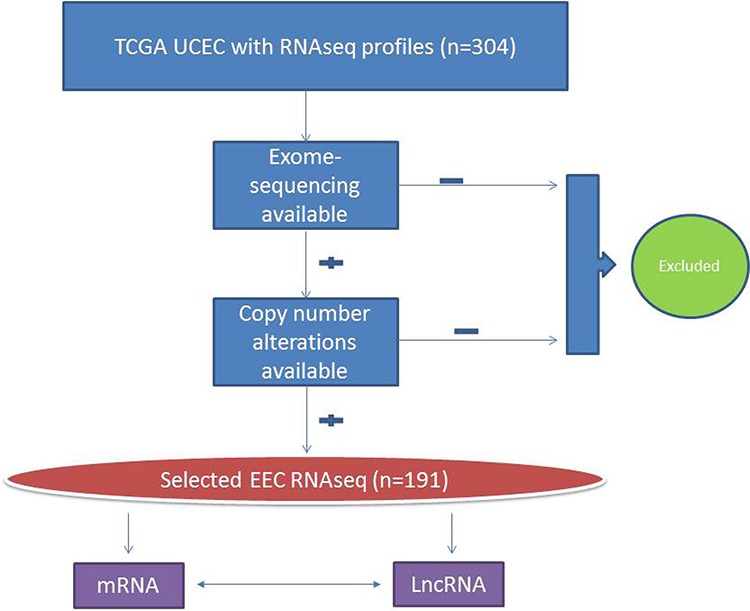
Flow chart showing the selection of endometrioid endometrial carcinoma cases from the Cancer Genome Atlas (TGCA) project Besides RNAseq, all cases with available exome-sequencing and copy-number data have been used as a training set. Cases without exome-sequencing and/or copy-number data have been used as a validation set for lncRNA classification.

In order to describe lncRNAs important in the development of EEC, we performed genomic analysis of lncRNAs using TCGA molecular RNAseq profiles of 191 primary tumors for which copy-number alterations and exome-sequencing data were available (Figure 1). According to GENCODE gene annotation v15, which represents the largest manually curated catalog of human lncRNA, the genome comprises 13,159 lncRNAs (i.e., lincRNA) categorized in seven categories as compared to 19,595 coding genes. To identify relevant lncRNAs, we first filtered the dataset to remove lncRNAs with low expression, defined as having an RPKM (reads per kilobase of transcript per million reads mapped) value <1 in at least 90% of the tumor samples. Using those criteria, we identified 1,931 lncRNAs that represent the potentially relevant lncRNAs in EEC (Supplementary Table 1). The expression of the majority of these lncRNAs was highly positively correlated (*r* > 0.33) with the expression of their neighboring genes; this is in contrast to as little as nine lncRNAs for which the expression was negatively correlated (*r* < −0.33) (Supplementary Table 1). Consistent with previous reports of EEC, we identified the expression of several known lncRNAs (i.e., *HOTAIR* and *H19)*, which gives us confidence in our methodology [10, 11]. Furthermore, we discovered the expression of novel lncRNAs not previously reported in EEC such as *HOTAIRM1, WT1-AS* and *MEG3* (Supplementary Table 1). Gene Ontology analysis using GREAT [12], which predicts the function of *cis*-regulatory regions, identified the gene set for the enrichment of histone H4 acetylation in an exclusive manner (*p* = 1.8 × 10^−5^; false discovery rate [FDR] = 0.003) (Supplementary Table 2). This set includes the two lysine histone acetyltransferase genes *KAT7* and *KAT8* as well as *MLL, EP400* and *EP300* genes.

We then asked if those 1,931 ECC-relevant lncRNAs have some extent of overlap with those in normal endometrium tissues. To do so, we generated the list of differentially expressed lncRNA between all EEC samples and 12 normal endometrium. Overall, 858 out of those (44.4%) were differentially expressed (FDR < 0.05) indicating the relevance of those lncRNA in endometrial carcinogenesis (Supplementary Table 3).

### Integrative analysis of lncRNA classification identifies basal-like, luminal-like and *CTNNB1*-enriched subgroups

**Figure 2:**
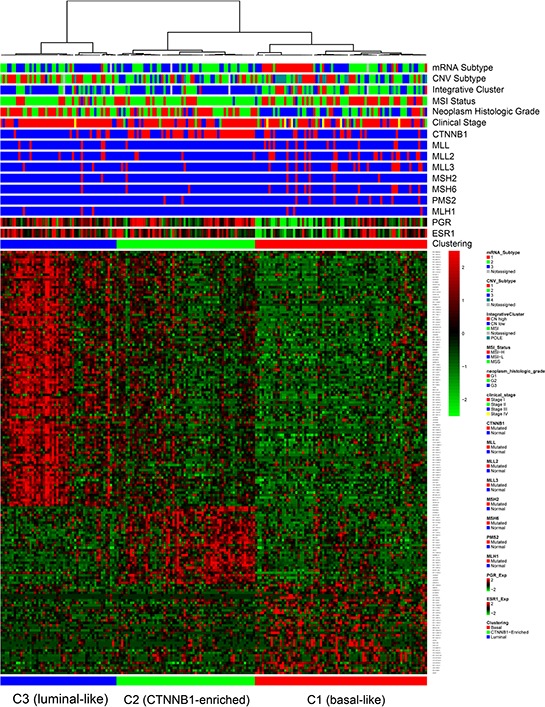
Hierarchical unsupervised clustering for endometrioid endometrial carcinoma cases in the training dataset, showing the presence of three molecularly distinct subgroups basal-like, CTNNB1-enriched and luminal-like.

We then performed integrative analysis in order to identify molecularly distinct tumor EEC subgroups associated with specific clinico-biological features. Unsupervised hierarchical clustering revealed three lncRNA subgroups that highly correlated with the mRNA transcriptomic classification (*p* < 0.0001 by Chi square test, and the Adjusted RAND Index is 0.4654), suggesting a crosstalk between lncRNA and mRNA (Figure 2).

**Figure 3:**
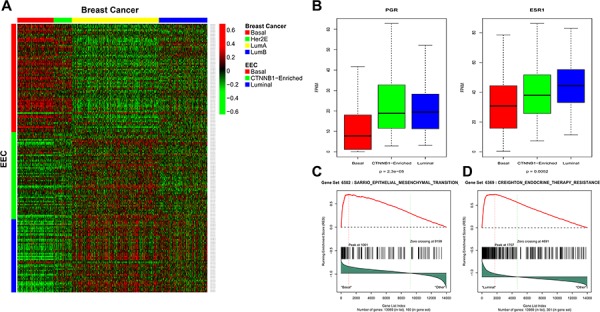
**A.** Heatmap for the concordance between endometrioid endometrial carcinomas (EECs) and breast cancer (BRCA) transcriptome classification. **B.** Box-plots for gene expression of progesterone (PGR1) and estrogen receptors (ESR1) in different EEC subgroups. **C.** Gene set enrichment analysis showing enrichment of epithelial–mesenchymal transition pathway in basal-like subgroup. **D.** Gene set enrichment analysis in luminal-like EEC subgroup showing enrichment of endocrine resistance therapy pathway in ESR1 and HER2 breast carcinomas.

Uterine serous endometrial carcinoma was previously shown to share genomic features with basal-like breast carcinomas [6]; therefore, we investigated whether our EEC lncRNA subgroup classification resembles the molecular classification of breast cancer. To answer this question, we performed supervised clustering using the PAM50 intrinsic subtype classification in a large dataset of breast tumors from TCGA, as previously reported [13]. Strikingly, we discovered that EEC cluster C1 was highly correlated with the basal-like breast cancer subtype. We thus named it the basal-like subgroup (Figure 3A) (Supplementary Table 4). In addition, cluster C3 was highly correlated with the luminal breast cancer subtype, thus we named it the luminal-like subgroup (Figure 3A). Importantly, the progesterone (PGR) and estrogen receptor (ESR1) genes showed low expression levels in the EEC basal-like subgroup as compared to the others (Figure 3B). Consistent with the basal-like subgroup classification, gene set enrichment analysis (GSEA) revealed that the EEC basal-like subgroup was positively enriched for the epithelial–mesenchymal transition (EMT) pathway (*p* < 10^−6^, FDR = 0.098) (Figure 3C). Of note, the luminal-like subgroup was enriched for the ‘group 2 set’ of genes associated with acquired endocrine therapy resistance in breast tumors that express *ESR1* and *ERBB2* (Figure 3D) (*p* < 10^−6^, FDR = 0.05), as reported by Creighton et al. [14]. To investigate pathways differentially regulated between cluster C2 and the other clusters, we performed GSEA and discovered that cluster C2 was exclusively enriched for signatures of hepatocellular carcinomas that harbor *CTNNB1* mutations in the two following datasets: BOYAULT_LIVER_CANCER_SUBCLASS_G6_UP (*p* < 10^−10^, FDR < 10^−6^) (Figure S1) and CHIANG_LIVER_CANCER_SUBCLASS_CTNNB1_UP (*p* < 10^−10^, FDR = 0.004) (Figure S2).

**Figure 4:**
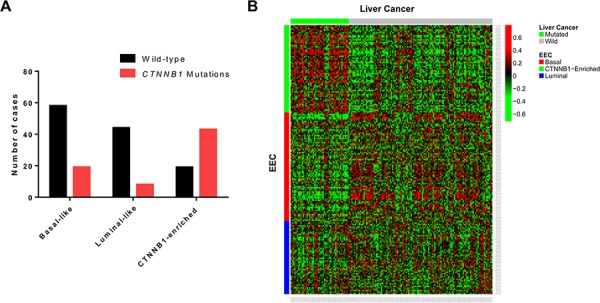
**A.** Distribution of CTNNB1 mutations in the three long non-coding RNA endometrioid endometrial carcinoma (EEC) subgroups. **B.** Heatmap for the concordance between EEC and liver cancer transcriptome classification.

We then analyzed the frequency of *CTNNB1* somatic mutations in the three EEC subgroups and confirmed enrichment for *CTNNB1* mutations in the C2 cluster. Overall, 69.3% (*n* = 43/62) of EEC samples in the C2 cluster harbored *CTNNB1* mutations as compared to 24.7% (*n* = 19/77) and 15.4% (*n* = 8/52) in the basal-like and luminal-like subgroups, respectively (*p* = 3.5 × 10^−10^) (Figure 4A). Using TCGA liver lncRNA dataset, we discovered a high correlation between EEC cluster 2 and the *CTNNB1*-mutated subclass of liver tumors (*p* = 3.1 × 10^−27^) (Figure 4B). We thus termed the C2 cluster as the *CTNNB1*-enriched subgroup.

As a result, our lncRNA classification of EEC is composed of 3 molecular subgroups: (1) basal-like, which is similar to the basal-like subtype of breast cancer, (2) *CTNNB1*-enriched, which is similar to *CTNNB1*-mutated subtypes of hepatocellular carcinomas, and finally (3) luminal-like, which is similar to the luminal-like subtype of breast cancer.

### Basal-like subgroup is enriched for aggressive tumors bearing alterations in polycomb and trithorax complexes

We then analyzed correlations between the three EEC subgroups and clinico-pathological tumor features. The basal-like subgroup (*n* = 77) was enriched for aggressive tumors that bear higher pathological grades (*p* < 0.0001) and higher FIGO stages (*p* = 0.01) (Supplementary Table 3). In addition, the basal-like subgroup was enriched for tumors classified in TCGA in the microsatellite instability and POLE subclasses (*p* < 0.0001) (Supplementary Table 3). Although we found no significant difference in the overall survival time of patients on the basis of the EEC subgroups, as assessed by Kaplan-Meier curves (Supplementary Figure S3A), we observed a trend toward worse survival in the basal-like subgroup as compared to the luminal-like subgroup (*p* = 0.06) (Supplementary Figure S3B).

**Figure 5:**
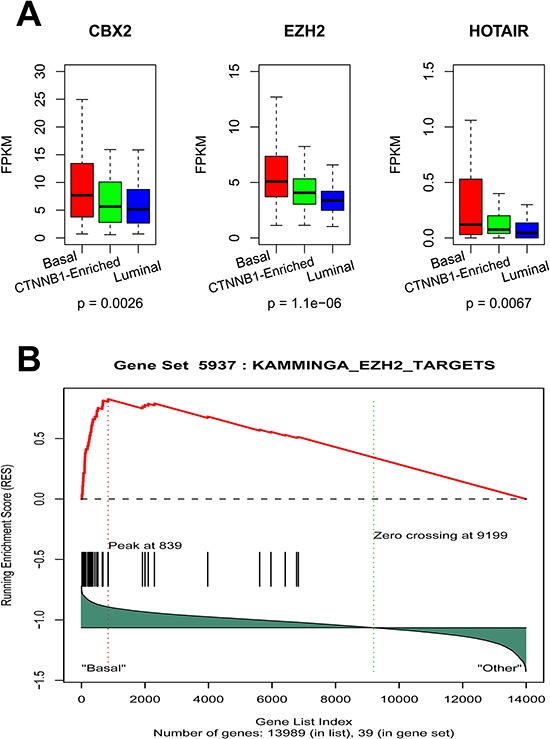
**A.** Box-plots for HOTAIR, EZH2 and CBX2 expression in the 3 lncRNA EEC subgroups. **B.** Gene set enrichment analysis revealing enrichment for EZH2 targets in the basal-like subgroup.

We then investigated the functional relevance of lncRNA differentially expressed between the EEC basal-like subgroup and the two other subgroups {fold-change (FC) ≥ 2 or ≤ 2, adjusted *p*-value < 0.05}. Overall, 33 lncRNAs were increased by more than two-fold and 50 lncRNAs were decreased by at least two-fold in the basal-like subgroup as compared to the two other subgroups (Supplementary Table 5). Interestingly, *HOTAIR* stood out as one of the top overexpressed lncRNAs (FC = 4; *p* = 0.0067; FDR = 0.01) in the basal-like subgroup (Figure 5A). Regarding the interplay between *HOTAIR* and the polycomb repressive complex (PRC), we then sought to determine whether any chromatin modifying genes (CMGs) are associated with our lncRNA subgroup classification. To do so, we manually curated a list of 161 CMGs and looked for differentially expressed genes in the three clusters. Strikingly, we identified 5 CMGs (*CTCFL, CBX2, ASF1B, HTLF* and *EZH2*) that were exclusively overexpressed in the basal-like subgroup (FC > 1.5, and adjusted *p*-value < 0.05), two in the luminal-like subgroup (*SATB1* and *RUVBL2*) and none in the *CTNNB1*-enriched subgroup. Interestingly, *CBX2* and *EZH2* genes, which were overexpressed in the basal-like subgroup (Figure 5A), are part of the PRC1 and PRC2 complexes, respectively. *CBX2* is thought to act on *EZH2* to activate PRC2, and thus the activity of both is important for polycomb gene silencing [15]. As *EZH2* was previously shown to bind to *HOTAIR*, we performed GSEA analysis, which pinpoints enrichment for *EZH2* targets within the EEC basal-like subgroup (KAMMINGA_EZH2_TARGETS, *p* < 0.0001; FDR = 0.05) (Figure 5B). We thus conclude that the basal-like EEC subgroup might be related to the activation of a subset of lncRNA that interacts with PRC1 and PRC2 through the *CBX2* and *EZH2* genes.

As progenitor cells which are located in the basal layer of human endometrium may express high levels of PRC2 as it is the case for other tissue types, we then asked whether the basal-like subgroup is enriched for signature of stem cells. Using GSEA, we found that WONG_EMBRYONIC_STEM_CELL_CORE signature was enriched in the basal-like subgroup as compared to others (Supplementary Figure S4A–S4B), suggesting that increased expression of the polycomb genes in basal-like subgroup may reflect increased fraction of cancer stem/progenitor cells in this setting. Likewise, GSEA analysis for differentially expressed lncRNA between basal-like EEC subgroup and normal endometrium reveals enrichment for WONG_EMBRYONIC_STEM_CELL_CORE (Supplementary Figure S4C–S4D). We conclude that basal-like subgroup may reflect increased fraction of cancer stem/progenitor cells as compared to other EEC subtypes as well as normal endometrium

### Pathway analysis of lncRNA subgroup classification

Using the upstream regulator functions of Ingenuity Pathway Analysis (IPA), we identified activation of the human chorionic gonadotropin complex (hCG) in the basal-like subgroup that is consistent with activation of the luteinizing hormone (LH)/hCG axis (Supplementary Table 6). Most of the aggressive EEC tumors that arise in postmenopausal women are apparently not linked to estrogen secretion. Thus, the basal-like subgroup might be sensitive to increased levels of LH/hCG. Of note, it has been shown that LH increases the invasiveness of human endometrial cancer cells through the activation of protein kinase A [16]; however, these results have been not conclusive in clinical findings [17].

Consistent with known *CTNNB1* mutations, IPA identified *CTNNB1* as the top altered pathway in the *CTNNB1*-enriched subgroup (Supplementary Table 7). Finally, the luminal-like subgroup was characterized by interleukin-22 (IL-22) activation (Supplementary Table 8). Importantly, IL-22 was demonstrated to promote proliferation of endometrial stromal cells [18].

### Association between somatic mutations and lncRNA subgroup classification

**Figure 6:**
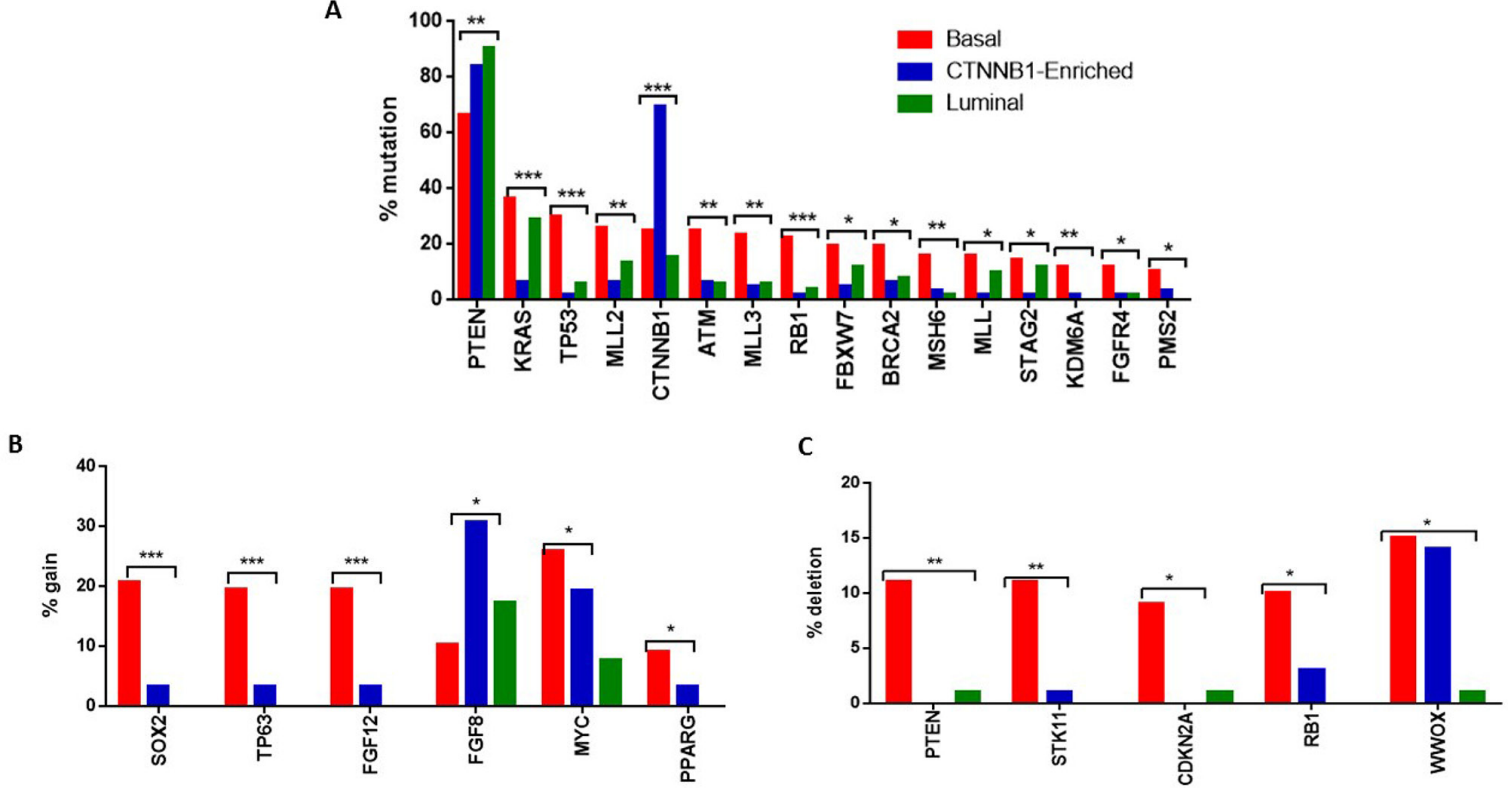
**A.** Bar graph showing the percentage of somatic mutations significantly altered between the three lncRNA subgroups. **B.** Bar graph showing the percentage of genes differentially and significantly gained between the three lncRNA subgroups. **C.** Bar graph showing the percentage of genes differentially and significantly deleted between the three lncRNA subgroups.

We then sought to determine whether the three lncRNA subgroups could define genetically distinct groups of EEC. To answer this question, we compared the prevalence of somatic mutations obtained through exome sequencing within the three clusters. Importantly, we found that the *CTNNB1*-enriched subgroup harbored frequent *PTEN* mutations but not for *KRAS* mutations, suggesting that *PTEN* loss but not *KRAS* mutations might enhance the *CTNNB1*-induced genetic program to promote tumor development in endometrial cancer (Figure 6A) (Supplementary Table 9). A *PTEN* loss results in the up-regulation of the PI3K-AKT-mTOR pathway [19]. Mutated *CTNNB1* expresses stabilized β-catenin, which cannot be phosphorylated by GSK3β [20], leading to further activation of the mTOR pathway. This likely explains the tumorigenesis mechanism in these individuals. This observation in the *CTNNB1*-enriched subgroup contrasts with the findings of a recent report, which indicated that either *KRAS* activation or *PTEN* loss similarly favored the dominant-stable *CTNNB1*-enriched genetic process and led to granulosa cell development in the ovary and testis [21]. However, the exclusion of *CTNNB1* and *KRAS* mutations was also observed in a small subset of patients with advanced EEC [22], indicating different tumorigenesis mechanisms. As expected, we identified frequent *TP53* mutations in the basal-like subgroup, which is consistent with an aggressive tumor. This was also identified in the serous-like endometrial cancer subtype according to mRNA classification [6]. Unexpectedly, we discovered that the basal-like EEC subgroup was characterized by frequent mutations in the trithorax group genes, encompassing the *MLL2* (26%), *MLL3* (23.4%) and *MLL* genes (15.6%) (Figure 6A) (Supplementary Table 9). Finally, the basal-like subgroup was also enriched for mutations in the H3K27 demethylase *KDM6A*, which is highly indicative of the key role of chromatin remodeling genes in establishing tumor aggressiveness in this subgroup.

### Association between copy number and lncRNA subgroup classification

We then looked for any association between copy number alterations and lncRNA subgroup classification. The basal-like subgroup displayed higher frequency of amplification of *SOX2, FGF12* and *TP63* genes as compared to the other two subgroups (Figure 6B). The *CTNNB1*-enriched subgroup was enriched for *FGF8* amplification as compared to the other subgroups (Figure 6B). We also found that the basal-like subgroup harbored frequent deletions of *PTEN, STK11, CDKN2A* and *RB1* (Figure 6C). Of note, *STK11* is a well-known tumor suppressor gene (Supplementary Table 10). A mouse model of non-small cell lung cancer tumors with *LKB1* (gene expression product of *STK11*) and *KRAS* mutation showed specific response to phenformin. Addition of the phenformin analog, metformin, in the combination of RAD001/letrozole also showed some response in a small subset of patients with advanced EEC in a phase II clinical trial [22]. Taken together, these findings suggest that *STK11*-related pathways can potentially be targeted in the subset of patients with *STK11* loss.

## DISCUSSION

We herein report for the first time that three subgroups of long non-coding RNA define the biological diversity of endometrioid endometrial carcinomas; of note, while basal-like and luminal-like EEC subgroups correlated well with breast carcinoma signatures, the *CTNNB1*-enriched subgroup correlated with *CTNNB1*-mutated liver carcinomas. These data are consistent with the systematic functional analysis of mRNA, in which hundreds of gene sets of diverse cancer profiles were analyzed and “modules” were defined as sets of genes for which gene expression is conditionally activated or repressed across a wide variety of cancer subtypes [23]. We thus conclude that lncRNAs act as modular scaffolds, allowing a higher order of ribonucleoprotein networks in chromatin states, which offers the opportunity to establish subgroups of cancers that arise within the endometrium [24].

To establish the lncRNA subgroup classification of EEC, we mined the dataset of TCGA to define the portrait of expressed lncRNA in this disease, which to our knowledge has not been described. Indeed, only a handful of lncRNAs has been reported so far as being expressed in EEC, such as *HOTAIR* and *CASC2* [10, 25]. Overall, we identified almost 2,000 expressed lncRNAs in EEC, representing 14.7% of GENECODE lncRNAs. Importantly, we discovered an enrichment of lncRNA located in the vicinity of genes involved in histone H4 acetylation. This is the case for *EP300*, which has been recently described to be mutated in 8% of serous endometrial carcinomas [26]. We thus conclude that in addition to the high rate of mutations in chromatin-remodeling genes (~36.5%) observed in serous endometrial carcinomas, deregulation of lncRNAs located in cis-regulatory elements (CREs) may also affect the expression of nearby genes involved in histone acetylation. Future studies are needed to establish the transcription factors that bind to these CREs and typically regulate gene transcription in EEC.

Another piece of evidence indicating the importance of epigenetics in the lncRNA classification of EEC was the discovery of the enrichment of the basal-like EEC subgroup with frequent mutations of trithorax and polycomb group genes. Of note, the basal-like subgroup was associated with aggressive clinico-pathological tumor features and poor outcome. Regarding polycomb complex alterations, we discovered overexpression of the methyltransferase *EZH2*, which is part of the PRC2 complex as well as the chromobox protein *CBX2*, which recruits PRC1 members to chromatin. To date, dysregulation of polycomb has been observed in many aggressive cancer subtypes but has not been linked to the basal-like EEC subgroup [27]. A recent report that investigated the expression of *CBX2* in human cancers showed initial evidence of an oncogenic role [28]; however, the overexpression of both *CBX2* and *EZH2* in the basal-like subgroup indicates the importance of interplay between PRC1 and PRC2 in endometrial cancer. These findings may facilitate the investigation of epigenetic therapy in this setting, as there are many EZH2 inhibitors in the early phase of clinical development.

Extensive evidence indicates that histone repressive proteins of the polycomb group recruit lncRNAs to the gene loci to silence their expression, which is the case for *HOTAIR* [9, 29]. Exploration of the entire spectrum of lncRNAs with functional interaction of the polycomb and trithorax groups will be a worthwhile future project. Within the basal-like EEC subgroup, we identified a core of lncRNAs that might, in addition to *HOTAIR*, silence the expression of their gene targets. Future mechanistic studies are needed to clarify the role of those lncRNAs. The underlying reasons for alterations within both trithorax genes that open the chromatin and polycomb genes that are involved in heterochromatin formation are not yet understood. We discovered *FGF8* amplification in the luminal-like EEC subgroup, suggesting that FGF inhibitors might represent therapeutic targets in this setting.

Finally, another topic that deserve also to discuss is the discovery of the *CTNNB1*-enriched EEC subgroup using lncRNA and which is consistent with the recent *CTNNB1*-enriched EEC transcriptomic subtype recently identified [30]. Of note, the majority of the cases displayed mutations in exon 3 which is similar to *CTNNB1* mutations identified in liver cancers. Future studies are needed to clarify the link between *CTNNB1* mutations and mRNA-lncRNA interplay in this subtype.

## MATERIALS AND METHODS

### The cancer genome atlas (tcga) data

endometrioid endometrial carcinoma (EEC) RNA-Seq data (BAM files) and their related clinical data were obtained from the Cancer Genomics Hub (CGHub, https://cghub.ucsc.edu/) and TCGA Data Portal (https://tcga-data.nci.nih.gov/tcga/). The list of cases is provided in Supplementary Table 11. The paired-end FASTQ files for each sample were extracted from BAM files using bam2fastq (http://www.hudsonalpha.org/gsl/information/software/bam2fastq). Both copy number alterations and somatic mutations were downloaded from The Broad GDAC Firehose http://gdac.broadinstitute.org/.

### Mapping/alignment

The raw paired-end (PE) reads in FASTQ format were aligned to the human reference genome, GRCh37/hg19, using MOSAIK alignment software [31]. MOSAIK works with PE reads from Illumina HiSeq and uses both a hashing scheme and the Smith-Waterman algorithm to produce gapped optimal alignments and to map exon junction-spanning reads with a local alignment option for RNA-seq. The resulting alignments were then saved as a standard BAM file.

We then counted the mapped reads in genomic features such as genes (mRNAs and lncRNAs) annotated in GENCODE15 to generate the raw counts for each gene using the HTSeq-count script distributed with the HTSeq package. We chose the “union” mode of HTSeq to mask the overlapping regions between mRNA and lncRNA to overcome the issue of non-strand-specific RNA sequencing KIT (TruSeq) in TCGA data.

### Count data normalization

Raw read count data were normalized across samples with DESeq_1.10.1 [32]. Specifically, DESeq first estimated the effective library size, which is also called size factor, by dividing each column by the geometric means of the rows given a matrix or data frame of raw count data. Then, the median of these ratios (skipping the genes with a geometric mean of zero) was used as the size factor for the column. With the estimation of size factors, DESeq then divided each column of the count table by the size factor for that column. That brought the count values to a common scale, making them comparable across samples. Furthermore, we transformed the count data by the varianceStabilizingTransformation function provided in the DESeq package. With this function, the standard deviation of each gene was roughly constant regardless of the gene expression magnitude.

### FPKM calculation

In our analysis, FPKM was calculated as the number of fragments per kilobase of non-overlapped exon per million fragments mapped. Since the raw count data per gene were generated with the “union” mode in HTSeq, whereby the reads mapped to the overlapping regions between mRNA and lncRNA were not counted, the exon sequences corresponding to the overlap between mRNA and lncRNA were excluded when we calculated the gene length for both mRNA and lncRNA.

### Low expression filtering

To reduce noise, we kept only mRNAs or lncRNAs with FPKM equal to or above 1 in at least 10% of samples for downstream analysis.

### Detection of differential mRNA and lncRNA expressions

All statistical analyses were performed using the R and R-Bioconductor statistical programming environment. We identified differentially expressed mRNAs and lncRNAs with DESeq, using the standard comparison mode between two experimental conditions. *P*-values were adjusted for multiple testing with the embedded Benjamini-Hochberg procedure in DESeq.

### Consensus clustering by lncRNAs

To assess the stability of the discovered clusters, we performed consensus clustering. Using the resampled data, we conducted 500 runs of hierarchical clustering. For each run, 80% of the samples and 80% of the lncRNAs were randomly chosen. The distance measurement was set as the Pearson correlation, and linkage function was set as “Ward”. Based on the 500 runs, a consensus was obtained by taking the average over the connectivity matrices of every perturbed dataset. Then we carried out hierarchical clustering with the consensus matrix as a similarity matrix, with “Euclidean” as the distance measurement and “Ward” as the linkage function. We also calculated the Bayesian information criterion to detect the number of clusters.

### Similarity heatmap between two different tumor types

We selected lncRNAs that are significantly overexpressed across the EEC subgroups and BRCA subgroups at FDR 0.05. Then FPKM values of these lncRNAs were log2 transformed (a constant 0.5 is added to FPKM values) and consequently converted to z scores in EEC and BRCA separately. A heatmap was generated from the Pearson correlation coefficients between EEC and BRCA samples on the lncRNAs selected.

## CONCLUSION

We report the first lncRNA subgroup classification of ECC, which has identified three lncRNA subgroups that are associated with different clinico-genomic features. The basal-like subgroup encompasses endometrial cancers with aggressive clinico-pathological features and correlates with frequent alterations of epigenetic regulators, including both mutations in trithorax group genes and overexpression of the two key polycomb genes, *EZH2* and *CBX2*. This application of lncRNA profiling can potentially be utilized as a tool for clinical outcome prediction and targeted therapy selection.

## SUPPLEMENTARY FIGURES AND TABLES




