# Supplementary material for: Long non-coding RNA profiling links subgroup classification of endometrioid endometrial carcinomas with trithorax and polycomb complex aberrations
Source: Oncotarget. 2015 Sep 26;6(37):39865–76. doi: 10.18632/oncotarget.5399 (PMC4741866; doi:10.18632/oncotarget.5399)
Supplement: Supplementary file 1 [file oncotarget-06-39865-s001.pdf]

SUPPLEMENTARY FIGURES AND TABLES

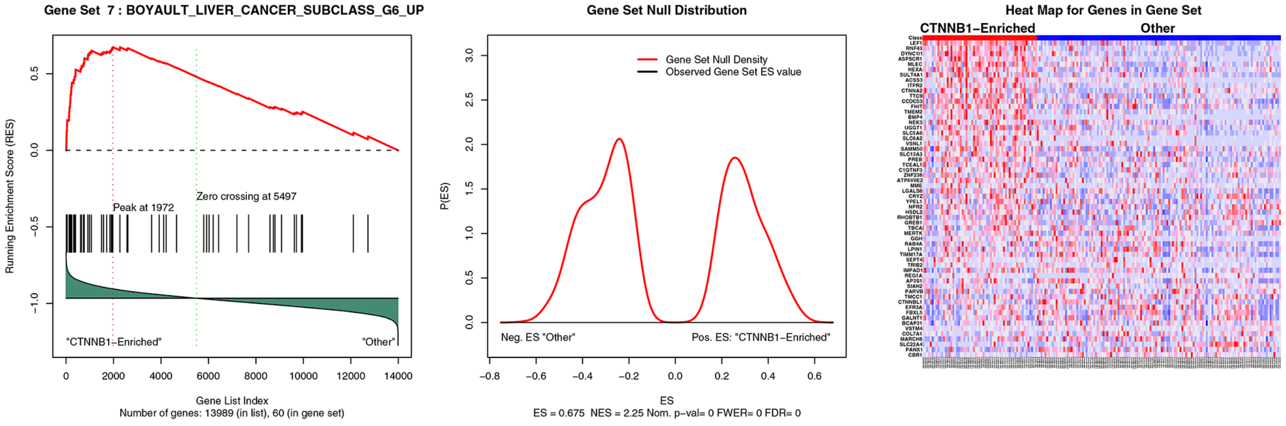

Supplementary Figure S1: Gene set enrichment analysis (GSEA) revealed that EEC CTNNB1-enriched subgroup was positively enriched for BOYAULT_LIVER_CANCER_SUBCLASS_G6_UP.

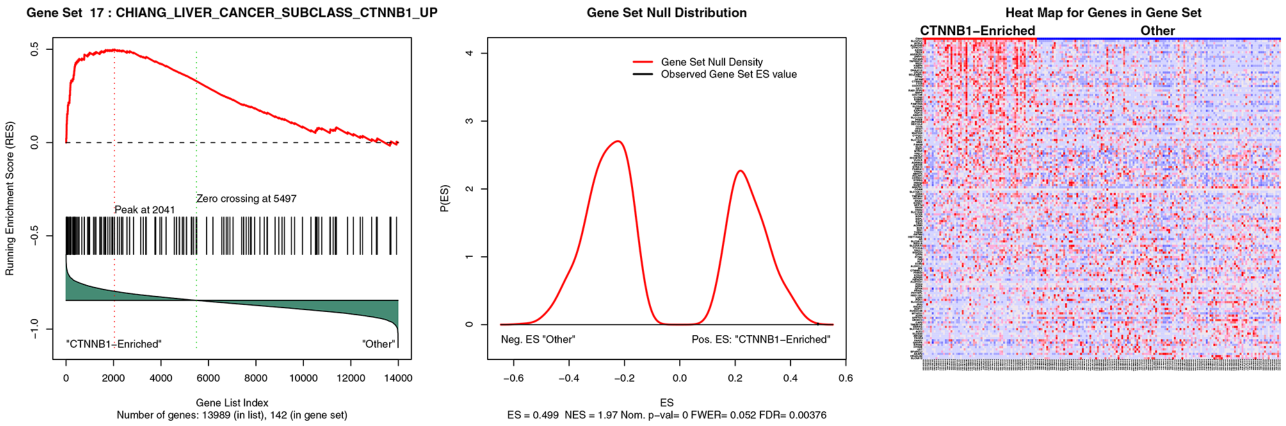

Supplementary Figure S2: Gene set enrichment analysis (GSEA) revealed that EEC *CTNNB1*-enriched subgroup was positively enriched for CHIANG_LIVER_CANCER_SUBCLASS_CTNNB1_UP.

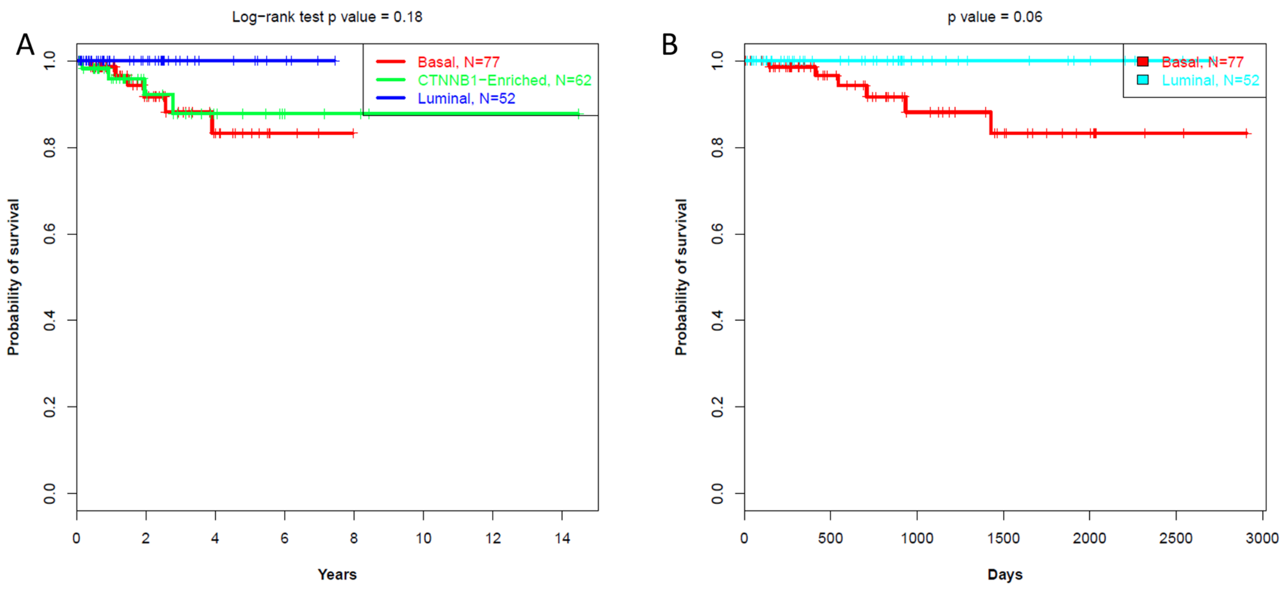

Supplementary Figure S3: A. Kaplan-Meier curves for overall survival times of patients according to the classification of three long-non-coding RNA subgroups of endometrioid endometrial carcinoma (EEC). **B.** Kaplan-Meier curves for overall survival times of patients according to their classification in basal-like and luminal-like EEC subgroups.

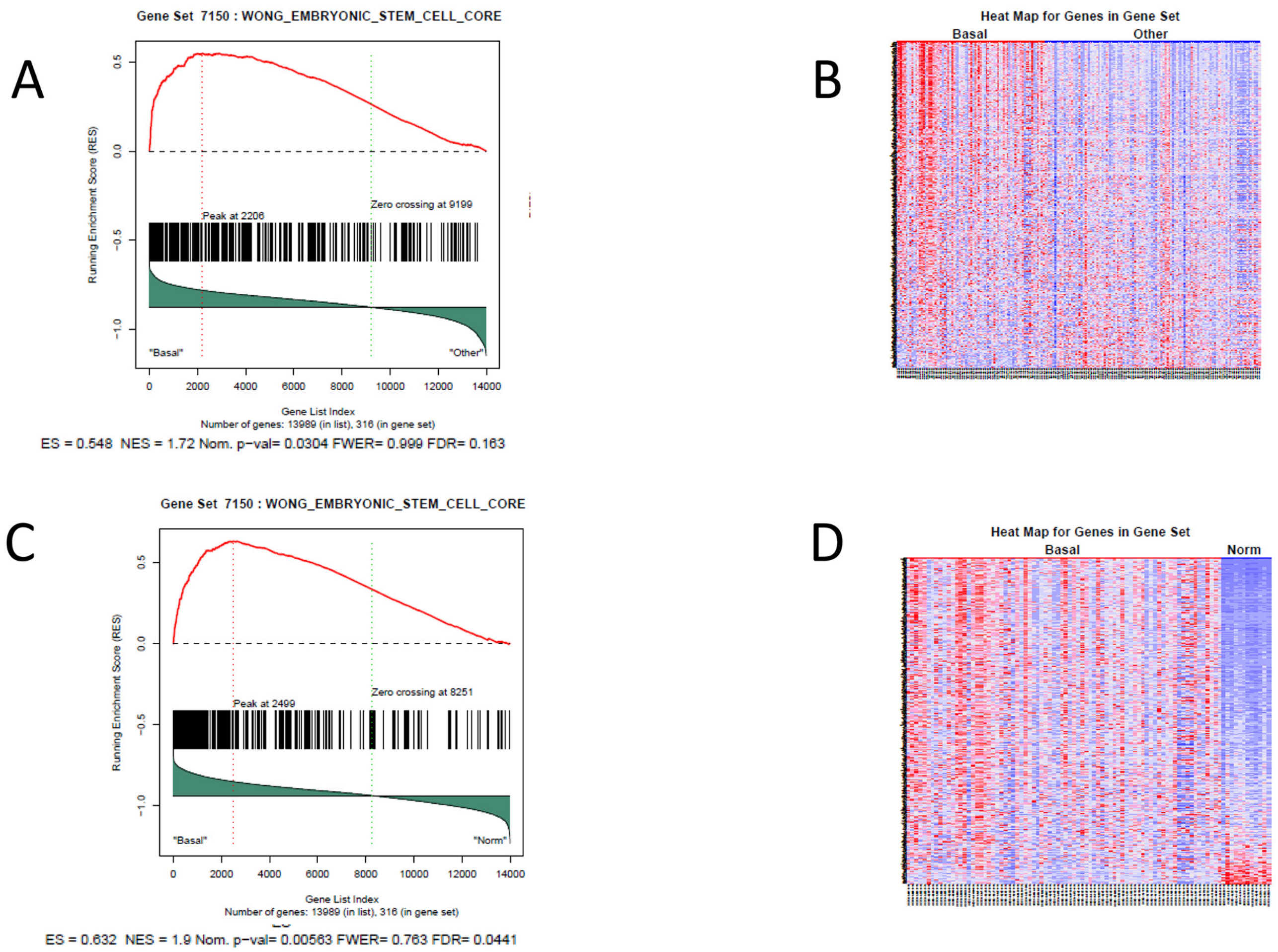

Supplementary Figure S4: A–B. Gene set enrichment analysis with relative heatmap showing enrichment for WONG_EMBRYONIC_STEM_CELL_CORE signature in basal-like subgroup of endometrioid endometrial carcinoma (EEC) as compared to other EEC subgroups. **C–D.** Gene set enrichment analysis with relative heatmap showing enrichment for WONG_EMBRYONIC_STEM_CELL_CORE signature in basal-like subgroup of EEC as compared to normal endometrium.

Supplementary Table S1: List of expressed long non-coding RNA (lncRNA) identified in the endometrioid endometrial carcinoma cases. Genes located in the vicinity of those lncRNA identified by GREAT are also reported. Correlation of the expression of each lncRNA with the expression of protein-coding genes located in the same vicinity is reported, in addition to the adjusted ***p***-value.

Supplementary Table S2: List of 14 genes belonging to histone H4 acetylation set identified by Gene Ontology analysis using GREAT, which predicts function of *cis*-regulatory regions located in the neighboring regions of long non-coding RNAs

**# GREAT version 2.0.2**	**Species assembly: hg19**
LncRNA	Neighboring gene
LEF1-AS1	LEF1 (-43224)
RP11–420A23.1	PHF17 (-403552)
RP11–215P8.3	PHF15 (-125399)
RP11–215P8.4	PHF15 (-81274)
AC004893.11	TRRAP (+152212)
RP11–135A24.4	EPC1 (+346)
TPBGL	ARRB1 (+109223)
CTD-2562J17.7	ARRB1 (+91255)
RP11–770J1.3	MLL (+85012)
RP13–820C6.2	EP400 (+129211)
RP11–196G11.4	KAT8 (+748)
RP11–640I15.1	CDRT1 (-33335)
AC087499.10	USP22 (+419966)
RP11–344E13.3	USP22 (+107685)
RP11–304F15.7	KAT7 (+46727)
AC005256.1	TCF3 (-98113)
RP1–85F18.5	EP300 (+93413)

Supplementary Table S3: Concordance between the long non-coding RNA subgroup classification of endometrioid endometrial carcinomas and other features such as mRNA classification, TCGA subtype classification and known clinico-pathological tumor features. And list of differentially expressed long non-coding RNA (lncRNA) identified in the endometrioid endometrial carcinoma cases as compared to normal endometrium. Abbreviation: FDR = False discovery rate.

Supplementary Table S4: Concordance between the long non-coding RNA subgroup classification of endometrioid endometrial carcinomas and PAM50 classification of breast cancer

	Basal	Her2E	LumA	LumB
Cluster 1 (Basal)	0	8.09E-212	8.03E-72	1.04E-256
Cluster 2 (CTNNB1-Enriched)	1	1	1	1
Luminal (luminal)	1	1	4.25E-296	6.25E-09

Supplementary Table S5: List of long non-coding RNAs differentially expressed between basal-like subgroup and the 2 other subgroups.

Supplementary Table S6: List of upstream regulators identified by Ingenuity Pathway Analysis (IPA) in the basal-like EEC subgroup as compared to the two other subgroups.

Supplementary Table S7: List of upstream regulators identified by Ingenuity Pathway Analysis (IPA) in the *CTNBB1*-enriched EEC subgroup as compared to the two other subgroups.

Supplementary Table S8: List of upstream regulators identified by Ingenuity Pathway Analysis (IPA) in the luminal-like EEC subgroup as compared to the two other subgroups

Upstream Regulator	Fold Change	Molecule Type	Predicted Activation State	Activation z-score	*p*-value of overlap	Target molecules in dataset
IL22		cytokine	Inhibited	−2,449	9,59E-06	CALML5,HP,IFNG,MUC4,MUC5B,SERPINA3
mir-34		microRNA	Inhibited	−2,000	2,60E-02	CCNE2,CPLX2,EMP1, KCNH2
ZNF217	0,308	transcription regulator	Inhibited	−2,000	8,84E-02	CCNE2,EPHX4,GAD1,HOXC6,LMO3,VSNL1
COL18A1	−0,195	other	Inhibited	−2,000	3,33E-01	EPHB1,IL6,ITGB3,MMP1
IL1A	0,950	cytokine	Activated	2,203	1,65E-07	CCL8,CXCL2,CXCL5,HSD11B1,IL11,IL6,LOX,MMP1,PDCD1LG2,PI3,PTX3,RHCG,S100A7,SERPINA3,TAC1,TNFRSF11B
Cg		complex	Activated	2,575	3,37E-03	BMP2,CCNE2,EMP1,FST,HMGA2,IL11,ITGB3,MCM10,MMP1,PKIA,PTX3
P38 MAPK		group	Activated	2,158	4,87E-03	CCL8,CCNE1,CXCL9,FST,HMOX1,IL6,ITGB3,MAL,MMP1,MMP3,MUC5AC,RRAD
PPRC1	0,078	transcription regulator	Activated	2,000	1,36E-01	AADAC,DUSP5,EPHB1, SPINK1

Supplementary Table S9: List of the frequency of the most frequently identified somatic mutations in the 3 long non-coding RNA subgroups of endometrioid endometrial carcinomas.

	Basal (*n* = 77)	CTNNB1-Enriched (*n* = 62)	Luminal (*n* = 52)	*p* value		Basal(%)	CTNNB1-Enriched(%)	Luminal(%)
CTNNB1_Mutated	19	43	8	3.55E-10	CTNNB1	24.68%	69.35%	15.38%
TP53_Mutated	23	1	3	8.72E-07	TP53	29.87%	1.61%	5.77%
KRAS_Mutated	28	4	15	6.09E-05	KRAS	36.36%	6.45%	28.85%
RB1_Mutated	17	1	2	6.73E-05	RB1	22.08%	1.61%	3.85%
MLL3_Mutated	18	3	3	0.001476	MLL3	23.38%	4.84%	5.77%
ATM_Mutated	19	4	3	0.00165	ATM	24.68%	6.45%	5.77%
PTEN_Mutated	51	52	47	0.002355	PTEN	66.23%	83.87%	90.38%
KDM6A_Mutated	9	1	0	0.004223	KDM6A	11.69%	1.61%	0.00%
MSH6_Mutated	12	2	1	0.006836	MSH6	15.58%	3.23%	1.92%
MLL2_Mutated	20	4	7	0.007252	MLL2	25.97%	6.45%	13.46%
MLL_Mutated	12	1	5	0.011944	MLL	15.58%	1.61%	9.62%
STAG2_Mutated	11	1	6	0.020419	STAG2	14.29%	1.61%	11.54%
FGFR4_Mutated	9	1	1	0.020986	FGFR4	11.69%	1.61%	1.92%
PMS2_Mutated	8	2	0	0.02165	PMS2	10.39%	3.23%	0.00%
FBXW7_Mutated	15	3	6	0.032917	FBXW7	19.48%	4.84%	11.54%
BRCA2_Mutated	15	4	4	0.04157	BRCA2	19.48%	6.45%	7.69%

Supplementary Table S10: List of the frequency of the most frequently identified gained and deleted genes in the 3 long non-coding RNA subgroups of endometrioid endometrial carcinomas

	Basal (n = 77)	CTNNB1-Enriched (n = 62)	Luminal (n = 52)	p value		Basal (n = 77)	CTNNB1-Enriched (n = 62)	Luminal (n = 52)
SOX2_Gain	16	2	0	2.20E-05	SOX2_Gain	20.78%	3.23%	0.00%
TP63_Gain	15	2	0	6.33E-05	TP63_Gain	19.48%	3.23%	0.00%
FGF12_Gain	15	2	0	6.33E-05	FGF12_Gain	19.48%	3.23%	0.00%
PTEN_Loss	9	0	1	0.002105	PTEN_Loss	11.69%	0.00%	1.92%
STK11_Loss	9	1	0	0.004223	STK11_Loss	11.69%	1.61%	0.00%
FGF8_Gain	8	19	9	0.010406	FGF8_Gain	10.39%	30.65%	17.31%
CDKN2A_Loss	7	0	1	0.01604	CDKN2A_Loss	9.09%	0.00%	1.92%
RB1_Loss	8	2	0	0.02165	RB1_Loss	10.39%	3.23%	0.00%
WWOX_Loss	12	9	1	0.022757	WWOX_Loss	15.58%	14.52%	1.92%
MYC_Gain	20	12	4	0.02667	MYC_Gain	25.97%	19.35%	7.69%
PPARG_Gain	7	2	0	0.048961	PPARG_Gain	9.09%	3.23%	0.00%

Supplementary Table S11: List of TCGA endometrioid endometrial carcinomas with related clinical information.
